# Sleep quality as a mediator between perceived stress and dry eye severity among Chinese nurses

**DOI:** 10.3389/fpubh.2026.1817895

**Published:** 2026-04-15

**Authors:** Jie Ren, Xin Zhang, Jia Li, Ke Ma

**Affiliations:** 1 Department of Ophthalmology, West China Hospital, Sichuan University, Chengdu, China; 2 West China School of Nursing, Sichuan University, Chengdu, China; 3 West China School of Medicine, Sichuan University, Chengdu, China

**Keywords:** dry eye, influencing factors, nurse, perceived pressure, sleep quality

## Abstract

**Aim:**

This study aims to investigate the prevalence and influencing factors of dry eye among Chinese nurses, and to explore the relationship between nurses’ perceived stress, sleep quality and dry eye.

**Methods:**

This study adopted a cross-sectional survey design and distributed electronic questionnaires through Wechat platform. A total of 450 valid questionnaires from nurses across the country were collected. The survey included demographic and sociological information on nurses, dry eye symptoms (Ocular Surface Disease Index), sleep quality (Pittsburgh Sleep Quality Index), and perceived stress (Perceived Stress Scale). Generalized linear model was used to analyze the factors affecting the symptoms of dry eye in nurses. The Process plug-in in SPSS was used to conduct mediation analysis using Bootstrap method.

**Results:**

Among the 450 nurses included, the prevalence of dry eye reached 66%. Specifically, mild dry eye accounted for 27.56%, moderate dry eye accounted for 14.89%, severe dry eye accounted for 23.56%. Multivariate analysis revealed that years in nursing, hospital grade, contact lens and frame glasses wearing, sleep quality and perceived stress were influencing factors for dry eye. Mediation analysis revealed that the bootstrap 95% confidence interval for the mediating effect of PSS score on OSDI score via PSQI score does not include zero.

**Conclusion:**

The prevalence of dry eye in Chinese nurses is very high, and the proportion of moderate to severe dry eye is relatively large. Years in nursing and wearing contact lenses or rimmed glasses were risk factors for dry eye in nurses. The perceived pressure and sleep quality are the direct risk factors of dry eye, and the perceived pressure can also influence the occurrence and development of dry eye through the mediating effect of sleep quality.

## Introduction

Dry eye is a chronic ocular surface disease resulting from multiple etiological factors, including environmental conditions (low humidity, air pollution, prolonged screen exposure), systemic diseases (Sjögren’s syndrome, rheumatoid arthritis, diabetes mellitus), hormonal changes (menopause, androgen deficiency), medications (antihistamines, antidepressants, isotretinoin), and anatomical abnormalities (lid malposition, meibomian gland dysfunction). These factors disrupt tear homeostasis through distinct pathophysiological mechanisms: tear film instability, hyperosmolarity, ocular surface inflammation, and epithelial damage with associated neural abnormalities (1). Clinically, dry eye manifests through a distinct set of symptoms and signs that affect both comfort and vision. Primary clinical manifestations include ocular irritation—such as redness, dryness, burning sensations, and foreign body sensation—as well as visual impairment, increased blinking frequency, and meibomian gland dysfunction. As the most prevalent ocular surface disorder, these symptoms significantly disrupt daily functioning (1). The burden of dry eye extends far beyond ocular discomfort, profoundly affecting patients’ quality of life, work productivity, and financial wellbeing (2). Research indicates that the impact of dry eye on health-related quality of life can be more substantial than that of severe ocular conditions such as glaucoma, macular degeneration, and retinal detachment (3). Furthermore, the reduction in quality of life reported by patients with moderate to severe dry eye is comparable to that experienced by individuals undergoing dialysis, suffering from severe angina, or coping with disabling hip fractures (4). Regarding prevalence, dry eye affects between 5 and 50% of the general population, with rates reaching as high as 70% in specific demographic groups (5). Looking forward, as China transitions into an aging society and the use of video terminals becomes increasingly pervasive in both professional and personal domains, the prevalence of dry eye is projected to rise further.

As healthcare professionals, nurses assume a critical role in the healthcare sector, dedicated to creating a secure healthcare environment and delivering high-quality services for patients (6). To ensure the smooth operation of the hospital and provide uninterrupted care for patients, nurses are required to work shifts (7, 8). Night shift work can disrupt nurses’ normal circadian rhythms (9), potentially leading to sleep disturbances and health-related issues. Research has indicated that hospital nurses experience the highest levels of occupational stress compared to other professions (10). When nurses are unable to effectively manage stress, the quality of care declines, the frequency of medical errors increases, and patient safety is threatened (11).

Bruce’s allostasis Theory posits that repeated or chronic exposure to stressors forces the neuroendocrine, cardiovascular, and immune systems into states of heightened variability or response. The cumulative “wear and tear” from these sustained responses, known as allostatic load, provides a critical physiological framework for understanding how chronic stress may compromise ocular surface homeostasis (12). Complementing this, the cognitive model of insomnia suggests that negative perceptual evaluations of stressful events trigger emotional distress and physiological arousal, which directly degrade sleep quality (13). Crucially, these theoretical concepts converge in the context of dry eye pathogenesis: the physiological arousal driven by allostatic load and the subsequent disruption of sleep quality can jointly impair tear secretion and destabilize the tear film (14). Thus, poor sleep acts not merely as a concurrent symptom but as a potential mechanistic mediator through which perceived stress exacerbates dry eye. Despite the established links between stress, sleep, and ocular health individually, the specific triadic relationship among perceived stress, sleep quality, and dry eye incidence remains underexplored, particularly within high-stress occupational groups. Nurses represent a critical population for this investigation due to their exposure to chronic shift work, high work pressure, and irregular sleep patterns, all of which are potent drivers of allostatic load. Consequently, this study aims to address this gap by investigating the current prevalence and influencing factors of dry eye among Chinese nurses. Specifically, it seeks to elucidate the interplay between nurses’ perceived stress and sleep quality, determining whether sleep quality serves as a mediating factor in the development of stress-related dry eye. These findings will provide a theoretical basis for developing targeted interventions to mitigate ocular surface disorders in healthcare professionals.

## Methods

### Participants and procedures

This study is a cross-sectional survey, utilizing questionnaires created via WeChat and the Questionnaire star software. The study participants completed the questionnaire through an online link. The questionnaire adopted standardized instructions and uniform filling guidelines to minimize the understanding biases of the research subjects. Inclusion criteria encompass: (1) Registered nurses who hold a valid nursing professional certificate and are currently engaged in clinical practice. (2) Nurses who voluntarily consent to participate in the research. Exclusion criteria encompass: (1) Nurses not involved in clinical work. (2) Nurses on leave for more than 3 months. According to the Kendall sample size estimation method, the recommended sample size is typically 5–10 times the number of variables, and considering sample loss and invalid questionnaires, with an additional expansion of 10–20%. In this study, there were 20 variables, leading to an estimated sample size ranging from 110 to 240 cases. This study strictly complied with the Declaration of Helsinki and approved by the Ethics Committee of the corresponding author’s hospital, with the approval number 2025 (36). All participants provided informed consent after being fully briefed on the study details and voluntarily agreed to participate.

A convenience sampling approach was used to recruit participants. The convenience sampling was conducted as follows: Step 1: We identified 40 hospitals that were willing to participate and had eligible staff members. Step 2: Study invitations were distributed through WeChat groups to all eligible individuals within these settings. Step 3: Interested participants who met the inclusion criteria voluntarily responded to the invitation. Step 4: Eligible participants who provided informed consent completed the questionnaires.

### Survey content

The questionnaire comprises two sections: an introductory section and the main questionnaire. The introduction outlines the purpose, content, and data usage, and emphasizes that participation in the survey is voluntary. The survey content included demographic information about nurses (such as age, years in nursing, frequency of night shifts per month, daily duration of mask-wearing, etc.). Additionally, it evaluated the severity of dry eye symptoms using the Ocular Surface Disease Index (OSDI), sleep quality through the Pittsburgh Sleep Quality Index (PSQI), and perceived stress via the Perceived Stress Scale (PSS). All the survey content has been uploaded to the “Questionnaire Star” platform^1^ and formatted into a distributable survey link.^2^ The link was subsequently disseminated via WeChat.

### Survey instruments

#### Ocular Surface Disease Index (OSDI)

The Chinese version of the Ocular Surface Disease Index was developed by Chen et al. (15), based on the original English version created by Allergan. The OSDI provides a simple, rapid, effective, and cost-efficient method for quantitatively assessing dry eye, enabling differentiation of varying severities of the condition. It enables the differentiation of dry eye severity by assessing ocular symptoms, visual-related functions, and environmental stimulus factors. The scale consists of 12 questions with scores ranging from 0 to 4 per question. The total score is calculated by multiplying the sum of all individual scores by 25 and then dividing the result by the number of questions answered. The total score ranges from 0 to 100, where higher scores indicate more severe symptoms (16–18). Classification criteria are as follows: ≤12 points indicate normal, 13–22 points indicate mild dry eye, 23–32 points moderate dry eye, 33–100 points indicate severe dry eye. The overall Cronbach *α* coefficient of the scale was 0.89 (15).

#### Pittsburgh Sleep Quality Index (PSQI)

The PSQI, developed by Dr. Buysse and colleagues at the University of Pittsburgh in 1989, assesses the overall sleep quality of participants over the past month. It is suitable for evaluating sleep quality among individuals with sleep disorders, psychiatric conditions, and for researching the relationship between psychological factors and sleep. The PSQI measures sleep quality across seven dimensions: subjective sleep quality, sleep duration, sleep latency, sleep disturbances, sleep efficiency, hypnotic drug use, and daytime dysfunction. Each dimension is scored from 0 to 3 points, resulting in a total score ranging from 0 to 21. A higher total score indicates poorer overall sleep quality. The score ranges are as follows: 0–5 points indicate excellent sleep quality, 6–10 points indicate decent sleep quality, 11–15 points indicate fair sleep quality, and 16–21 points indicate poor sleep quality (19). In China, a 7-point threshold is commonly employed to delineate sleep quality scores, where a score of ≥7 indicates poor sleep quality, with a sensitivity of 98.3% and specificity of 90.2% (20).

#### Perceived Stress Scale (PSS)

The Perceived Stress Scale was originally developed by American scholar Dr. Sheldon Cohen (21) in 1983 and subsequently adapted into Chinese by Professor Yang Tingzhong. The Chinese version of this scale has demonstrated satisfactory reliability and validity among Chinese nursing professionals (22). The scale is an internationally recognized and widely utilized instrument for measuring pressure. It comprises 14 items that reflect the sensations of tension and perceived loss of control associated with stress, and it requires respondents to provide answers based on their personal experiences. The scale is divided into five levels ranging from “Never” to “Most of the time” with values from 0 to 4. Items 4, 5, 6, 7, 9, 10, and 13 are reverse-scored and pertain to the dimension of tension. Items 1, 2, 3, 8, 11, 12, and 14 are positively scored and belong to the dimension of perceived loss of control. The total score ranges from 0–56 points, where a score below 29 indicates normal pressure, 29–42 indicates high pressure, and over 42 indicates excessive pressure. Higher scores correspond to greater levels of pressure. The overall Cronbach’s *α* is 0.882, indicating good reliability and validity.

### Statistical method

Data analysis was performed using IBM SPSS Statistics for Windows, Version 26.0 (IBM Corp., Armonk, N. Y., USA). For quantitative data, descriptive statistics employ means and standard deviations, while qualitative categorical data are represented through percentages (constituent ratios). Pearson correlation analysis was employed to examine the relationships between continuous variables (age, years of nursing experience, frequency of night shifts per month, daily duration of mask wearing, work-related video terminal exposure time, leisure-related video terminal exposure time, total video terminal exposure time, PSQI scores, PSS scores, and OSDI scores). The assumptions of linearity, bivariate normal distribution, and the absence of significant outliers were checked using scatter plots and Q–Q plots prior to analysis. A Chi-square test was conducted to examine differences in OSDI scores across various working departments, hospital grades, and spectacles-wearing conditions. The assumption of sufficient sample size was met, ensuring that the expected frequency in each cell was greater than 5. Subsequently, a generalized linear model was employed to integrate significant variables identified in the univariate analysis into the multivariate analysis. The model assumptions regarding the normality of residuals and homoscedasticity were evaluated. Mediation analysis was conducted using the Process plug-in in SPSS, employing the Bootstrap method (5,000 resamples) to estimate the 95% confidence intervals for the indirect effects. This method is robust and does not require the assumption of normality for the mediating effect.

## Results

The survey garnered a total of 486 responses from nurses across the country. A questionnaire was considered unqualified and thus excluded from the final analysis if it met any of the following criteria: ① Incomplete responses: Questionnaires with a significant amount of missing data, specifically, those with more than 20% of items unanswered or with key variables left blank. ② Straight-lining patterns: Responses that showed no variance, where the participant selected the same option for all questions within a multi-item scale, indicating a lack of careful consideration. ③ Unreasonably short completion time: Questionnaires completed in a time that was less than half of the median completion time, suggesting that the participant did not read the questions carefully. ④ Inconsistent answers: Questionnaires containing contradictory responses to logically related questions or failed attention-check items. After excluding unqualified questionnaires, 450 valid responses were included in the analysis, resulting in an effective response rate of 92.59%.

### Descriptive statistical analysis

Means, standard deviations, and proportions (component ratios) were utilized to describe the quantitative and qualitative categorical data, respectively, as presented in Tables 1, 2. The participants’ ages had a mean of 35.33 ± 7.28 years, with a range of 23–55 years. Among them, only 153 nurses had normal OSDI scores. Therefore, the prevalence of dry eye among nurses in this study was 66%.

**Table 1:** Descriptive statistics for continuous variables.

Variables	Mean	Standard deviations
Age	35.33	7.28
Years in nursing	13.64	7.86
Frequency of night shifts per month	4.02	3.89
Duration of mask-wearing (h/day)	6.94	2.75
Work-related video terminal exposure time (h/day)	5.03	2.07
Leisure-related video terminal exposure time (h/day)	4.96	2.51
Total video terminal exposure time (h/day)	9.99	3.68
OSDI score	21.62	18.15
Sleep quality	1.19	0.81
Sleep latency	1.41	0.90
Sleep duration	1.35	0.86
Sleep efficiency	0.54	0.91
Sleep disturbances	1.18	0.64
Hypnotic drug use	0.33	0.83
Daytime dysfunction	1.57	0.96
PSQI score	7.57	3.88
Perceived loss of control	11.77	4.68
Sensations of tension	12.30	5.29
PSS score	24.07	6.83

**Table 2:** Descriptive statistics for categorical variables.

Variable	Frequency	Percentage (%)
Work department		
Internal medicine	85	18.89
Department of surgery	211	46.89
Outpatient service	38	8.44
Operating room	35	7.78
Others^*^	81	18.00
Hospital grade		
Tertiary grade A hospital	355	78.89
Tertiary grade B hospital	57	12.67
Others^#^	38	8.44
With chronic disease		
Yes	76	16.89
No	374	83.11
Glasses wearing condition		
Wear contact lenses	59	13.11
Wear glasses	133	29.56
No	258	57.33
History of ophthalmic surgery		
Yes	67	14.89
No	383	85.11
OSDI classification		
Normal	153	34.00
Mild dry eye	124	27.56
Moderate dry eye	67	14.89
Severe dry eye	106	23.56
PSQI classification		
Excellent sleep	153	34.00
Decent sleep	199	44.22
Fair sleep	82	18.22
Poor sleep	16	3.56
PSS classification		
Normal	333	74.00
High pressure	116	25.78
Excessive pressure	1	0.22

* Others include critical care, specialty and other departments.

# Others include second class and other hospital grades.

### Univariate analysis of OSDI among nurses

The Pearson correlation coefficient was employed to examine the relationships between age, years in nursing, frequency of night shifts, duration of mask wearing, work-related video terminal exposure time, leisure-related video terminal exposure time, total video terminal exposure time, PSQI score, PSS score, and OSDI score. Univariate analysis revealed significant correlations between the OSDI score and several factors, including the frequency of night shifts, duration of mask wearing, work-related video terminal exposure time, leisure-related video terminal exposure time, total video terminal exposure time, PSQI score, and PSS score. The results are presented in Table 3.

**Table 3:** Univariate analysis of continuous variables and OSDI.

Variable	Pearson correlation coefficient	*p*-value
Age	0.02	0.65
Years in nursing	0.05	0.29
Frequency of night shifts per month	0.10	**0.03**
Duration of mask-wearing (h/day)	0.10	**0.03**
Work-related video terminal exposure time (h/day)	0.12	**0.01**
Leisure-related video terminal exposure time (h/day)	0.22	**0.00**
Total video terminal exposure time (h/day)	0.22	**0.00**
PSQI score	0.48	**0.00**
PSS score	0.35	**0.00**

Bold values indicate statistical significance at *P* < 0.05.

Chi-square tests were conducted to examine differences in OSDI scores across various working departments, hospital grade, and glasses-wearing conditions. Independent samples *t*-tests were employed to assess whether significant differences existed in OSDI scores between individuals with and without chronic diseases, as well as those with and without a history of ophthalmic surgery. The findings indicated that OSDI scores were significantly associated with work department, hospital grade, presence of chronic disease, and glasses-wearing status. The results are presented in Table 4.

**Table 4:** Univariate analysis of categorical variables and OSDI.

Variable	OSDI score Mean ± Standard deviation	Test statistic	*p*-value
Work department			
Internal medicine	23.25 ± 19.75	2.79	**0.03**
Department of surgery	23.83 ± 19.26		
Outpatient service	17.89 ± 14.46		
Operating room	17.62 ± 14.97		
Others	17.64 ± 15.17		
Hospital grade			
Tertiary grade A hospital	21.90 ± 18.60	3.24	**0.04**
Tertiary grade B hospital	16.86 ± 15.09		
Others	26.19 ± 16.90		
With chronic disease			
Yes	27.55 ± 19.95	2.90	**0.01**
No	20.42 ± 17.55		
Glasses wearing condition			
Wear contact lenses	24.87 ± 18.51	5.19	**0.01**
Wear glasses	24.75 ± 21.10		
No	19.26 ± 16.02		
History of ophthalmic surgery			
Yes	20.07 ± 16.12	−0.76	0.45
No	21.89 ± 18.49		

Bold values indicate statistical significance at *P* < 0.05.

### Multiple regression analysis of OSDI among nurses

In this study, a generalized linear model was employed to integrate significant variables identified in the univariate analysis into a multivariate analysis framework. (Note: Given the strong collinearity between the total video terminal exposure time and both work-related and leisure-related video terminal exposure time, the total time has been excluded from the multi-factor analysis. Additionally, based on professional considerations, years in nursing are deemed relevant to OSDI score, and thus this variable has also been included in the analysis.) The multivariate linear regression model was constructed using basic information, PSQI scores, and PSS scores as independent variables, with OSDI scores serving as the dependent variable.

The OSDI score exhibited a gradual increase with the rise in year in nursing, PSQI score, and PSS score. Specifically, for each additional year in nursing, the average OSDI score increased by 0.23 points (*p* = 0.03). A one-point increase in the PSQI score was associated with an average increase of 1.67 points in the OSDI score (*p* = 0.00). Similarly, a one-point increase in the PSS score corresponded to an average increase of 0.54 points in the OSDI score (*p* = 0.00). Contact lens wearers exhibited an average OSDI score that was 6.37 points higher than non-wearers (*p* = 0.01). Individuals who wore glasses demonstrated an average OSDI score that was 5.54 points higher compared to those who did not wear glasses (*p* = 0.00). The results are presented in Table 5.

**Table 5:** A multiple regression analysis of OSDI (*n* = 450).

Variables	Regression coefficient		Standardized regression coefficient	t	*p*
	*β*	Std. Err			
Intercept	−15.74	4.65	-	−3.39	**0.00**
Years in nursing	0.23	0.11	0.10	2.15	**0.03**
Frequency of night shifts per month	0.14	0.23	0.03	0.63	0.53
Duration of mask-wearing (h/day)	0.35	0.27	0.05	1.28	0.20
Work-related video terminal exposure time (h/day)	0.35	0.38	0.04	0.93	0.35
Leisure-related video terminal exposure time (h/day)	0.42	0.32	0.06	1.32	0.19
PSQI score	1.67	0.21	0.36	7.88	**0.00**
PSS score	0.54	0.12	0.21	4.72	**0.00**
Work department (with outpatient service as the control group)					
Internal medicine	−1.58	3.37	−0.03	−0.47	0.64
Department of surgery	0.21	3.02	0.01	0.07	0.95
Operating room	−2.82	3.66	−0.04	−0.77	0.44
Others	−5.12	3.29	−0.11	−1.56	0.12
Hospital grade (with tertiary grade A hospital as the control group)					
Tertiary grade B hospital	−1.56	2.46	−0.03	−0.63	0.53
Others	5.42	2.73	0.08	1.99	0.05
With chronic disease (with no as the control group)					
Yes	1.45	1.99	0.03	0.73	0.47
Glasses wearing condition (with no as the control group)					
Wear contact lenses	6.37	2.28	0.12	2.79	**0.01**
Wear glasses	5.54	1.70	0.14	3.26	**0.00**

Regression model testing: *F* = 12.95, *p* < 0.001; Goodness of fit: coefficient of determination = 0.32.Bold values indicate statistical significance at *p* < 0.05.

Multicollinearity: The analysis showed that all VIF values were well below the standard threshold of 5 (range: 1.08–4.42). The Tolerance values were all greater than 0.2. These results indicate that there is no serious multicollinearity among the variables. The results are presented in Table 6.

**Table 6:** Coefficients.^a^

Mode	Unstandardized coefficients		Standardized coefficients	*t*	Sig.	Collinearity statistics	
	*B*	Std. error	Beta			Tolerance	VIF
(Constant)	−15.74	4.65		−3.39	0.00		
Years in nursing	0.23	0.11	0.10	2.15	0.03	0.70	1.42
Frequency of night shifts per month	0.14	0.23	0.03	0.63	0.53	0.66	1.53
Duration of mask-wearing (h/day)	0.35	0.27	0.05	1.28	0.20	0.92	1.08
Work-related video terminal exposure time (h/day)	0.35	0.38	0.04	0.93	0.35	0.86	1.17
Leisure-related video terminal exposure time (h/day)	0.42	0.32	0.06	1.32	0.19	0.81	1.24
PSQI score	1.67	0.21	0.36	7.88	0.00	0.76	1.31
PSS score	0.54	0.12	0.21	4.72	0.00	0.83	1.21
Work department—internal medicine	−1.58	3.37	−0.03	−0.47	0.64	0.30	3.38
Work department—department of surgery	0.21	3.02	0.01	0.07	0.95	0.23	4.42
Operating room	−2.82	3.66	−0.04	−0.77	0.44	0.54	1.87
Work department—others	−5.12	3.29	−0.11	−1.56	0.12	0.32	3.10
Hospital grade—tertiary grade B hospital	−1.56	2.46	−0.03	−0.63	0.53	0.77	1.30
Hospital grade—others	5.42	2.73	0.08	1.99	0.05	0.89	1.12
Chronic disease—yes	1.45	1.99	0.03	0.73	0.47	0.92	1.08
Glasses wearing condition—wear contact lenses	6.37	2.28	0.12	2.79	0.01	0.86	1.16
Glasses wearing condition—wear glasses	5.54	1.70	0.14	3.26	0.00	0.86	1.17

^a^Dependent variable: OSDI score.

### Mediation effect analysis of PSS and OSDI

**Figure 1:**
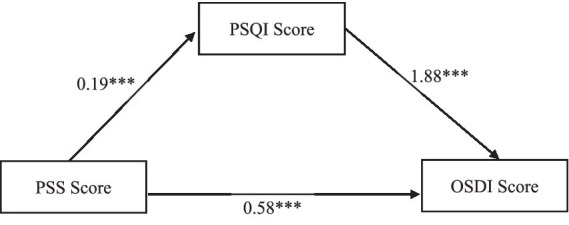
Path coefficients of PSS, PSQI, and OSDI. ^***^p < 0.001.

Univariate analysis revealed significant associations between PSS and PSQI with OSDI, suggesting the potential existence of an intermediary relationship. Mediation analysis was conducted using the Process plug-in in SPSS, employing the Bootstrap method. The path coefficients among the three variables are illustrated in Figure 1.

As illustrated in Table 7, the bootstrap 95% confidence interval for the mediating effect of PSS score on OSDI score via PSQI score does not include zero. This indicates that PSS score not only has a direct effect on OSDI score, but also has a mediating effect on OSDI score through the variable PSQI score. Specifically, the direct effect (0.58) and the indirect effect (0.36) constitute 61.70 and 38.30% of the total effect (0.94), respectively.

**Table 7:** The mediation effect analysis of OSDI.

Effect Type	Effect value	SE	95%CI		Proportion (%)
			Lower	Upper	
Total effect	0.94	0.11	0.71	1.17	–
Direct effect	0.58	0.11	0.36	0.81	61.70
Indirect effect	0.36	0.07	0.23	0.50	38.30

## Discussion

### Current situation and demographic influencing factors of dry eye in Chinese nurses

In this study, which included 450 nurses, the prevalence of dry eye was found to be 66%. Specifically, 124 nurses (27.56%) experienced mild dry eye (12 < OSDI ≤ 22), 67 nurses (14.89%) had moderate dry eye (22 < OSDI ≤ 32), and 106 nurses (23.56%) suffered from severe dry eye (32 < OSDI ≤ 100). This suggests that the prevalence of dry eye among Chinese nurses is significantly high, exceeding that observed in German residents (21.6%) (23) and Chinese internet professionals (50.8%) (24). In addition to the special nature of the work, the higher prevalence of dry eye among nurse practitioners in China may also be associated with the predominantly female composition of this profession, as gender is a recognized risk factor for the condition (25). The findings were lower compared to a Turkish survey reporting a 70.5% prevalence of dry eye among operating room nurses (26). This phenomenon may be attributed to alterations in blinking frequency and patterns resulting from nurses’ prolonged concentration on tasks in the operating room, as well as the impact of intense lighting, harsh chemicals, and surgical smoke in the work environment (26). Majority of the participants were from surgical department, and this was followed by medicine and other departments (comprising critical care, specialty and others). In our study, we found a prevalence of dry eye among nurses of 66%. This finding is consistent with the trend reported by Riyad (27), which observed a rise in prevalence from 54.0% pre-pandemic to 62% post-pandemic among healthcare workers. The increased incidence of dry eye can be attributed to reduced outdoor activities, prolonged use of video terminals, and extended periods of mask-wearing (27). This suggests that engaging in more outdoor activities during their leisure time may contribute to nurses maintaining optimal eye health.

Aging is frequently recognized as a significant risk factor for dry eye, as cellular senescence may result in diminished tear production with advancing age (28). However, in this study, age was not identified as a significant factor influencing the prevalence of dry eye. This observation may be attributed to the relatively narrow age range of the study participants (average age was 35.33 ± 7.28 years, with a range of 23–55 years.), who were predominantly middle-aged and younger working nurses, thus not encompassing older adults or perimenopausal individuals. The incidence of dry eye in women has been shown to correlate with hormonal levels, which typically fluctuate more prominently during perimenopause, thereby increasing the prevalence of dry eye in that demographic (29).

Multivariate analysis showed that the OSDI score increased gradually with the increase of years in nursing, and the average OSDI score increased by 0.23 points (*p* = 0.03) for each additional year of nursing years. A large-sample study identified occupational factors as significant contributors to dry eye. Individuals working prolonged hours in indoor, air-conditioned environments while facing video terminals show a higher prevalence of the condition compared to those in outdoor, physically demanding roles (30). For nurses, extended work duration directly increases exposure to these risk-prone environments. Furthermore, Allostasis Theory (12) suggests that beyond environmental triggers, chronic work-related stress and load may also constitute significant risk factors for dry eye. However, general hospital nurses often have limited awareness of the importance of ocular health and tend to underestimate eye-related symptoms (31), resulting in nurses paying insufficient attention to the prevention and symptom management of dry eye. This also suggests that ophthalmology healthcare professionals can carry out dry eye-related educational programs in the hospital to improve the dry eye related knowledge of health care workers, including nurses.

Multifactorial analysis revealed that contact lens wearers exhibited an average OSDI score 6.37 points higher than non-wearers (*p* = 0.01). Additionally, nurses who wore glasses demonstrated an average OSDI score 5.54 points higher than those who did not wear glasses (*p* = 0.00). Prior research has indicated that wearing contact lenses can elevate the prevalence of dry eye, primarily due to their potential impact on tear film stability, which may contribute to the development of dry eye (27). This study found that nurses wearing frame glasses had higher OSDI scores. This finding aligns with the emerging concept of Mask-Associated Dry Eye (MADE) (32). As described by Moshirfar (32), face masks can direct exhaled breath upwards towards the ocular surface, increasing the rate of tear evaporation and disrupting the tear film stability. Glasses may act as a physical barrier that traps the exhaled air escaping from the top of the mask, creating a localized “wind tunnel” effect or preventing the dissipation of the humid air layer over the eye. This constant exposure to exhaled air accelerates tear evaporation more aggressively than the mask alone. The non-significance of mask-wearing duration may indicate that the presence of this altered airflow dynamic (exacerbated by glasses) is the primary driver of symptoms, rather than a linear accumulation of exposure time. Therefore, the combination of mask-wearing and glasses usage creates a specific high-risk microenvironment for dry eye, distinct from the effects of mask duration alone. Research has found that health care workers wearing protective glasses at work can ease the symptoms of dry eye (33). Because the protective glasses can form a closed space with the face when worn, the protection of the eyes is similar to the wet room mirror, while avoiding the acceleration of air flow caused by breathing when wearing a mask. Therefore, when conditions allow, medical personnel can choose to wear protective glasses at work rather than ordinary frame glasses or contact lenses in order to mitigate the symptoms of dry eyes.

### The relationship between sleep quality, stress perception and dry eye

Multivariate analysis showed that OSDI score increased with the increase of PSQI and PSS score. Furthermore, the mediation effect analysis found that PSS score not only had a direct effect on OSDI score, but also had a mediating effect on OSDI score through PSQI score, the direct effect (0.58) and the mediating effect (0.36) constituted 61.70 and 38.30% of the total effect, respectively.

Studies have found that psychological stress can increase pain by inducing elevated cortisol levels (34). Chronic stress not only indirectly promotes inflammation through glucocorticoid receptor resistance, but also activates inflammatory signaling pathways such as STAT-3 and NF-κB, further aggravating the inflammatory microenvironment (34). Dry eyes are often accompanied by ocular surface inflammation (1). Thus, stress perceived by nurses is not only a direct risk factor for dry eye, it can also make the symptoms of dry eye felt by individuals more severe. Several studies have reported the relationship between sleep quality and dry eye (35, 36). The impact of sleep on tear production can be elucidated through two primary mechanisms. Firstly, parasympathetic tone is thought to stimulate tear secretion, and reduced sleep duration leads to diminished parasympathetic activity, thereby decreasing tear secretion (37). Secondly, inadequate sleep may disrupt the secretion of hormones associated with dry eye, such as androgens (14). Therefore, the deterioration in sleep quality among nurses can reduce tear production and lead to dry eye. Previous research has demonstrated a significant association between high levels of stress and insomnia (38). Consequently, the stress perceived by nurses may not only directly increase the risk of dry eye but also indirectly contribute to reduced tear secretion through its adverse effects on sleep quality, leading to dry eye. This suggests that we can take some intervention methods (such as sleep intervention (39), auricular point therapy (40), etc.) to reduce the psychological pressure of nurses and improve their sleep quality, so as to achieve the purpose of effectively controlling dry eye.

The lack of significance for “night shifts” and “VDT time” in the model likely reflects a mediation effect. Night shifts and prolonged VDT use are upstream stressors that disrupt circadian rhythms and increase systemic stress. Our data suggests that these factors exert their influence on dry eye symptoms primarily through the deterioration of sleep quality and the elevation of psychological stress. Once the model accounts for the direct impact of PSQI and PSS, the residual direct effect of the environmental stressors becomes statistically non-significant.

## Conclusion

The prevalence of dry eye among Chinese nurses is notably high, with a considerable proportion experiencing moderate to severe symptoms. Factors such as years in nursing and wearing contact lenses or glasses have been identified as risk factors for dry eye. Perceived stress and sleep quality are direct risk factors for dry eye, while perceived stress can also indirectly influence the onset and progression of dry eye through the mediating effect of sleep quality.

### Limitations

Our study had several limitations. First, it was a cross-sectional design and was inherently limited in its inability to confirm the cause-and-effect relationship between individual variable and dry eye of Chinese nurses. While our analysis suggests that psychological factors (PSS) and sleep quality (PSQI) are strong predictors of OSDI scores, an alternative explanation—reverse causality—must be considered. Therefore, the relationship observed in our cohort likely represents a complex bidirectional feedback loop rather than a unidirectional cause-effect pathway. Longitudinal studies with repeated measures are necessary to clarify the temporal precedence of these factors and to determine whether psychological interventions can break this cycle in patients with DED. Longitudinal studies are required to further clarify these relationships. Second, influencing factors were self-reported by volunteers, which might have induced recall bias. Nevertheless, this can be considered as an inherent limitation of any survey-based study. Moreover, the sample size included in this study was small, whereas the uneven distribution of demographic characteristics among the participants makes confirming the significance of the results difficult.

## Data Availability

The raw data supporting the conclusions of this article will be made available by the authors, without undue reservation.

## Footnotes

1
https://www.wjx.cn/

2
https://www.wjx.cn/vm/etaBOO5.aspx
